# The evolving roles of US political partisanship and social vulnerability in the COVID-19 pandemic from February 2020–February 2021

**DOI:** 10.1371/journal.pgph.0000557

**Published:** 2022-12-05

**Authors:** Justin Kaashoek, Christian Testa, Jarvis T. Chen, Lucas M. Stolerman, Nancy Krieger, William P. Hanage, Mauricio Santillana

**Affiliations:** 1 Harvard College, Cambridge, Massachusetts, United States of America; 2 Machine Intelligence Group for the Betterment of Health and the Environment, Network Science Institute, Northeastern University, Boston, Massachusetts, United States of America; 3 Department of Social and Behavioral Sciences, Harvard T.H. Chan School of Public Health, Boston, Massachusetts, United States of America; 4 Department of Pediatrics, Harvard Medical School, Boston, Massachusetts, United States of America; 5 Department of Mathematics, Oklahoma State University, Stillwater, Oklahoma, United States of America; 6 Department of Epidemiology, Harvard T.H. Chan School of Public Health, Boston, Massachusetts, United States of America; The University of British Columbia, CANADA

## Abstract

The COVID-19 pandemic has had intense, heterogeneous impacts on different communities and geographies in the United States. We explore county-level associations between COVID-19 attributed deaths and social, demographic, vulnerability, and political variables to develop a better understanding of the evolving roles these variables have played in relation to mortality. We focus on the role of political variables, as captured by support for either the Republican or Democratic presidential candidates in the 2020 elections and the stringency of state-wide governor mandates, during three non-overlapping time periods between February 2020 and February 2021. We find that during the first three months of the pandemic, Democratic-leaning and internationally-connected urban counties were affected. During subsequent months (between May and September 2020), Republican counties with high percentages of Hispanic and Black populations were most hardly hit. In the third time period –between October 2020 and February 2021– we find that Republican-leaning counties with loose mask mandates experienced up to 3 times higher death rates than Democratic-leaning counties, even after controlling for multiple social vulnerability factors. Some of these deaths could perhaps have been avoided given that the effectiveness of non-pharmaceutical interventions in preventing uncontrolled disease transmission, such as social distancing and wearing masks indoors, had been well-established at this point in time.

## Introduction

Over the course of 2020, multiple interventions aimed at mitigating the spread of COVID-19 became highly politicized amid the hyper-partisan environment of the United States in an election year. While local political partisanship may have been and still be an important contributor to the effectiveness of pandemic management, few studies to date have explored its role beyond anecdotal observations [1–4]. In order to better understand how political partisanship may influence local pandemic response, we propose considering other possible important contributing factors including but not limited to racial demographics, income, education, and population density.

There has been extensive evidence documenting the inequities in risk of exposure and death among communities whose members are predominantly low-income and/or Black, Hispanic, or American Indian/Alaska Native [5–9], and the roles of various non-pharmaceutical interventions in reducing morbidity and mortality while vaccines were unavailable [10–15]. Numerous works have pointed to the importance of population density [16] or income [9, 17], but these factors may have contributed differently as the pandemic evolved in the US. Much of these previous studies in the United States have been conducted on the state level and examine regional differences or differences within a specific state [6, 8].

Works that have shown the importance of behavioral variables, while often controlling for certain variables such as race/ethnicity, do not comprehensively analyze behavior, socio-economic, and vulnerability factors together. Works that analyze the effects of the disease on less granular levels may also be misleading because they ignore the extreme heterogeneous impact of the pandemic. Finally, it is clear that factors that are associated with a rapid COVID-19 surge in cases in New York City and the Northeast earlier in 2020 are unlikely to be exactly the same that are associated with the early Fall surge in the Midwest or the eventual nationwide third wave that peaked in January 2021.

As we will show, the first year of the pandemic in the United States was marked by three distinct phases: an initial spread through major cities and coastal regions (February–May 2020), secondary spread through the South and rural areas of the Northeast and Mid-Atlantic (June–September 2020), and saturation of all remaining areas, mostly in the West (October 2020–February 2021). We study the relationship between county-level COVID-19 deaths and a collection of social, demographic, political factors, and vulnerability variables associated with each phase of the pandemic. In our analysis, we include political partisanship (quantified as county-level political lean toward the Democratic or Republican 2020 presidential candidate) and party affiliation of state governors, given their role in approving or forbidding state-level mandates for mask-wearing and other COVID-19 statewide policies. We select variables to include in our analysis based on the availability of reliable county-level data sets and the existence of academic or intuitive justification.

Our goal is to understand and describe the disparities that are related to the pandemic locally across the US, in particular over its most intense period –in terms of reported deaths– between October 2020 and February 2021. This is an important task in both responding to imminent future infectious disease emergencies (including any response to emerging COVID-19 variants of concern, such as Delta and Omicron) and also guiding the ongoing response elsewhere (including vaccination efforts).

## Materials and methods

### Data

#### COVID-19 deaths data

We collected county-level COVID-19 attributable death counts, at a daily resolution, from the Center for Systems Science and Engineering at Johns Hopkins University’s publicly available data repository [18]. This variable was chosen as the response variable throughout our analysis. For comparability purposes, we standardized the time series after applying a 7-day average to remove (day-of-the-week)reporting biases. We limited our data to the 50 U.S. states and Washington D.C.

#### Political leaning data

We obtained county-level data on the outcome of the 2020 presidential elections from the New York Times [19]. We calculate political leaning as the difference between the number of votes in a county in favor of the Republican party and the number of votes in favor of the Democratic party in the 2020 election divided by the county’s population. A value of -1 would indicate a county where everyone voted for the Republican party in the most recent election (and a value of 1 would indicate a county where all votes went to the Democratic party).

#### Population variables

We obtained data on median household income, race/ethnicity (percentage of the population who are Black, only, and percentage of the population who are Hispanic), household crowding, and population density from the American Community Survey’s 2019 5-year estimates [20]. Household crowding is the estimation of the number of households with more people than rooms. For each of these variables, we split counties into one of five quantiles.

#### Mandate stringency

We split counties in a given quantile of a population variable into three tiers according the stringency of their mandates in a given period: lax, moderate, and strict. The stringency of mandates is determined by the Oxford COVID-19 Government Response Tracker (OxCGRT) [21]. We only include mandates that are imposed at the state-wide level.

### Methods

#### Clustering

**Fig 1:**
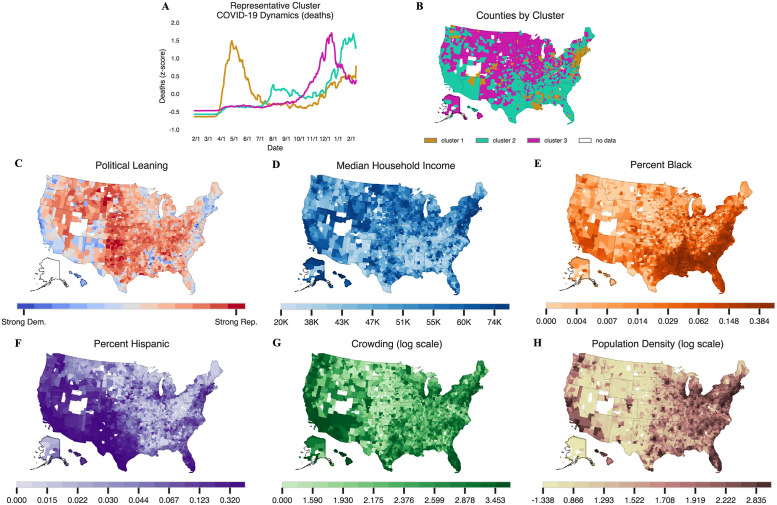
A summary of 3-means clustering and maps of counties included in the analysis shaded by the six different population variables of interest. (**B)** A map of cluster assignments for all US counties with data and at least 1 death. Northeastern counties and counties surrounding New Orleans largely comprise the first cluster, while southern counties generally comprise the second cluster, and the remaining counties comprise the third cluster. (**C-H)** show counties shaded according to six different population and socio-economic variables. (**G**) and (**H**) show the log of crowding and population density, respectively. Counties without any color are those with missing data. Counties are plotted using the U.S. Census Bureau’s 2019 shapefiles [24].

In order to characterize the dynamics of COVID-19 mortality in the U.S. during the first year of the pandemic, we first performed *k*-means clustering on the temporal (normalized) trajectories of the death data at the county-level. We found *k* = 3 to be both a parsimonious and meaningful choice for the number of clusters (see Fig 1 and Section “Clustering” in S1 Text for details). These clusters had a distinct peak, which allowed us to split the year into three time periods: February to May 2020, June to September 2020, and October 2020 to February 2021.

#### Bivariate analysis

In each of these three periods, we conducted a bivariate analysis where we examined correlations between death rates and one of six county-level variables (political leaning, median household income, Black residents (percent), Hispanic residents (percent), household crowding, and population density), separated by stringency of state-level mandates.

#### Multi-factorial analysis

To further investigate whether or not any of the associations identified in our clustering and bivariate analyses persisted when incorporating additional social vulnerability and other human behavior proxies, we built multiple models where the response variable is the severity of a local outbreak (mortality) and the predictors (or input variables) consist of a diverse set of variables of interest, shown graphically on S1 Fig [30]. Due to the sparsity of mask-wearing data, we include results for models that included these data in S6 Fig. For more details on the variables included and their data sources, see S1 Text.

In what follows, we distinguish between early introduction of the virus and death “severity” attributable to COVID-19 in a county.

#### Virus introduction

First, a county must be exposed to the virus, which we call virus introduction. We say that virus introduction occurs once a county has seen at least 5 COVID-related deaths, and we build models to predict whether a county is introduced to the virus in a given period using a logistic regression approach [22].

#### Virus transmission severity

Second, we quantify the intensity at which the virus spread through a county (deaths per 100,000), which we call virus severity. To predict COVID-19 severity, we build models to investigate which potential predictors best explain the death rate in a county. We implement a suite of models that allow us to be confident in the best predictors of virus spread in each time period. The results that are presented in the main manuscript are the outputs from both a regularized multivariate linear regression –that uses the least absolute shrinkage and selection operator (LASSO)– and a regularized implementation of a random forest regression. The second of these was built to better account for nonlinear effects in our modeling framework, although we do allow for Box-Cox transformation for the predictors in our LASSO model. Taking a “regularized” approach allows us to identify and remove redundancies –potential high positive correlations– between the socio-economic and behavioral variables to better characterize the effects of the multiple variables in this study. Regularization also allows us to constrain the coefficients on our variables to prevent over-fitting. Since we normalized the values of the predictors (to range from 0 to 1), the coefficient values associated with each of our explanatory variables in the linear regression models quantify their importance, and their sign determines whether they are associated with higher mortality (positive) or lower mortality (negative). In contrast, random forest regressions allow us to identify the importance of features as predictors –via permuted feature importance– but not the sign of their contribution.

The other models that we implemented, the results of which are presented in the supplement, include a conditional auto-regressive (CAR) Poisson model and a spatial lag model. We implement a Poisson model because these are more often used on count data (such as death counts). Although we attempted to account for spatial autocorrelation (the idea that counties that are closer together will have similar death rates due to their proximity to each other) in the linear regression and random forest regression by including the latitude and longitude of the center of a county as possible predictors, the spatial lag model includes a lag term that better accounts for the spatial structure of the data.

In the severity analysis for the third time period, we allow prior infections to be predictors of severity (higher deaths) by incorporating three additional variables as input: previous deaths (Black), previous deaths (Hispanic), and previous deaths (all races/ethnicities), calculated as the proportion of a county’s Black, Hispanic, and total population who died in periods 1 and 2 (COVID-19 racial data was obtained from The COVID Tracking Project [23]). The intuition behind this choice is that it allow us to identify if a county experiences many (or few) deaths in period 3 because they experienced very few (or many) deaths in periods 1 and 2.

In each model, we manually and in a step-wise fashion, remove variables that are highly correlated with each other (see S1 Text for more details on how we selected which variables to remove).

## Results

We first provide our clustering results to motivate our cutoffs for each of the three periods and examine a breakdown of COVID-19 deaths in those periods by census region and political leaning. We then present our bivariate analysis, incorporating the stringency of governor interventions. Finally, we present our multi-factorial analysis where we robustly analyze the predictive power of variables of interest.

### Clustering: The trajectory of the pandemic varied substantially by region and rurality

Fig 1A and 1B show the clustering results that justify the use of three clusters. As shown in Fig 1A, counties in cluster 1 were those which peaked early in the spring of 2020, recovered in the summer, and saw a graduate uptick in the late fall. Inspecting Fig 1B, these counties tend to be located along the Boston-Washington D.C. corridor in the Northeast, with additional pockets around Detroit, Chicago, New Orleans, and Seattle. These counties tend to either be urban or are adjacent to urban areas. However, not all urban counties are part of cluster 1. For example, metropolitan areas in California, the Southwest, Texas, and Florida are not part of this cluster.

These metro areas do appear in cluster 2, however, along with almost the entire South, rural New England, the more populated parts of Texas and Oklahoma, and all but the most remote parts of the West Coast, as shown in Fig 1B. Cluster 2 counties are characterized by a summer peak, though much less dramatic than cluster 1. Unlike the counties in cluster 1, however, these counties never recovered. Case rates dip slightly in October and November, only to spike dramatically to unprecedented levels in December 2020 and January 2021.

The remaining counties fall into cluster 3, characterized by a single but severe peak, occurring in December just as the other clusters started their second surge. However, by the time clusters 1 and 2 had reached their second peaks around the end of January, the cluster 3 counties had substantially recovered, with COVID-19 rates falling to half their peak levels (See Fig 1B). These counties include some of the most rural parts of the country—Alaska, west Texas, the Rocky Mountain West, and the Dakotas—but large Midwestern metro areas such as Milwaukee and Minneapolis are also included. Rural counties in New York, Pennsylvania, Ohio, and Illinois also fall into this late-breaking cluster despite their relative proximity to urban centers of cluster 1; in some cases, the two clusters are even adjacent.

**Fig 2:**
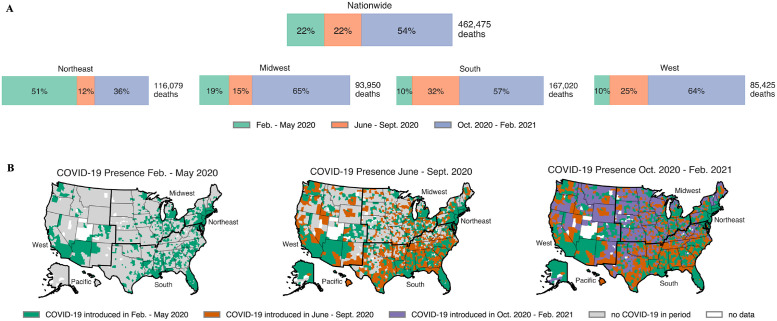
A breakdown of COVID-19 presence across the periods of interest. (**A**) In the top half of the figure, we present the percentage of deaths in the nation by period, both nationwide and by Census region. Other than the Northeast, which was hit hard in the first period, the nation was hit hardest in period 3, as pointed out in [25]. This fact motivates a closer lens on period 3. (**B**) COVID-19 onset at the county level. A county is treated as infected once it has experienced at least 5 COVID-related deaths. We see the movement of COVID from the cities and coastal areas to the center of the county over the course of the year. Counties and regions are plotted using the U.S. Census Bureau’s 2019 shapefiles [24].

Using the date ranges determined in our clustering analysis, we examine deaths in each of the three periods (Fig 2). In terms of geography and total number of deaths during these three periods, Fig 2 shows that the pandemic first took hold in the Northeast and major cities (See green counties on the left map on panel B) before spreading to the South in period 2 (orange counties on the center map on panel B). By period 3, the disease had taken hold across the nation, and the Midwest, South, and West all experienced most of their COVID-19 related deaths. The COVID-19 mortality rates in each of the nation’s 3,243 counties (This includes county equivalents such as boroughs in Alaska, parishes in Louisiana, and the District of Columbia. We employ “county” as shorthand to refer all of the above.) followed a unique trajectory during the pandemic. Some were infected earlier but peaked later, some experienced multiple peaks, and so forth.

**Fig 3:**
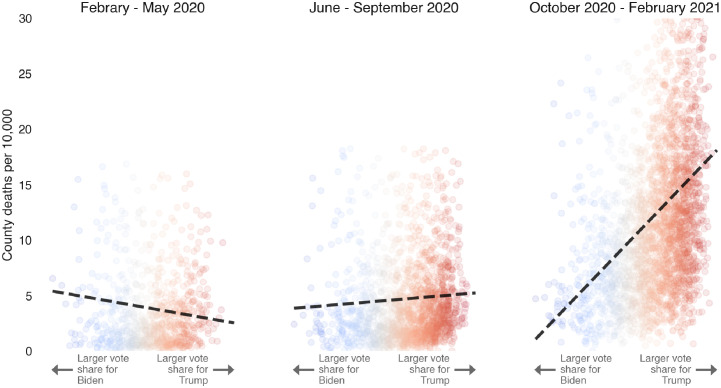
Evolution of COVID-19 deaths vs political leaning. COVID-19 attributed deaths (per 10,000) at the county level as a function of vote share in favor of J. Biden (Democratic) vs. D.J. Trump (Republican), 2020 presidential candidates, during the three periods of interest. The dashed black line on each figure indicated a line of best fit. Inspiration for this figure comes from David Leonhardt’s New York Times article, “Red COVID” [26].

Fig 3 shows a particularly important trend that we focus on and will robustly test: Democratic-leaning counties were hit slightly more strongly than Republican-leaning ones during the early months of the pandemic, but by the third period, deaths in Republican-leaning counties were 2–3 times higher than in Democratic-leaning counties.

### Bivariate analysis: Counties in states with stringent state-wide social distancing mandates experienced less severe COVID-19 death rates

**Fig 4:**
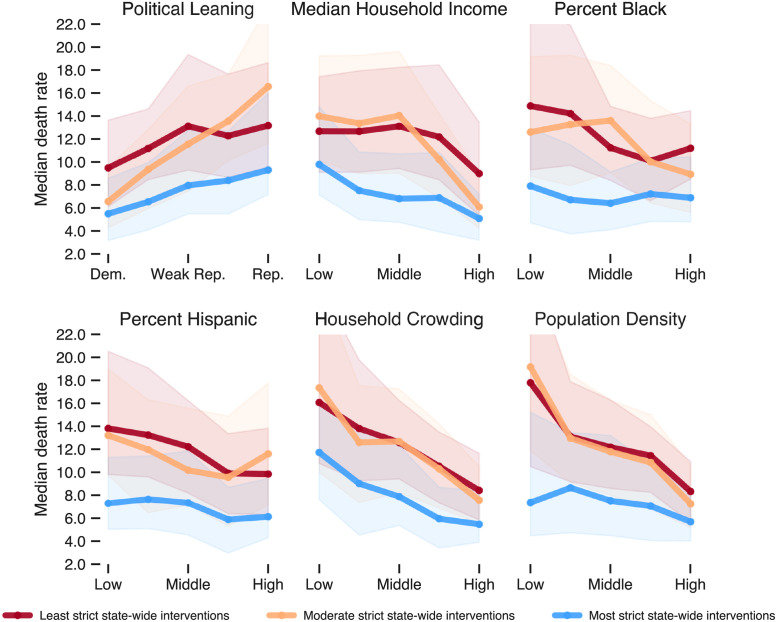
Median death rate in period 3 for each of the six sociodemographic variables broken down by the stringency of state mandates. For each variable, counties are grouped into quintiles. For instance, in the first plot, the leftmost three points represent the median death rate for the 20% of counties with the highest Democratic vote share in lax-, moderate-, and strict-mandate states, respectively. Shaded bands represent the IQR. The differences in each vertically stacked set of three medians are different at the *p* < 0.001 level (Mood’s median test).

One potential confounder that we must control for is the stringency of state-wide mandates. For instance, the states that chose to impose stricter mandates may have been those whose populations were naturally most at risk. In our first attempt to do so, we conduct a bivariate analysis, the results of which are shown in Fig 4 and S2 Fig. Fig 4 focused only on the third period in our analysis when deaths in Republican-leaning counties were the highest.

As shown in all six plots of Fig 4, for period 3, the median death rate in counties with the strictest mandates (blue lines) is consistently lower than counties in the moderate and lax tiers, no matter which sociodemographic variable is being considered. The differences between the tiers are all significant at the p<0.001 level using a Mood’s median test for each set of three medians shown. Interestingly, there is not much difference between counties in the moderate or lax tiers.

Although states with Republican governors were more likely to impose more lax social distancing mandates, Republican presidential vote share still appears correlated with county death rate in all three tiers (Fig 4, top left plot). That is, within a given state, we see that counties that voted overwhelmingly for Trump have a higher mortality rate than those that voted overwhelmingly for Biden, even though both were subject to the same mandates. Household crowding and population density continue to correlate with mortality within states, although that correlation is negative during this period. The relationship between race and death rates is more ambiguous in this period; the percentage of Black or Hispanic residents in a country does not make much difference if strict mandates are in place. In states where mandates were moderate or lax, heavily Black and Hispanic counties suffered higher mortality.

### Multi-factorial analysis: Republican-leaning counties in states with lax mandates experience the most deaths

**Fig 5:**
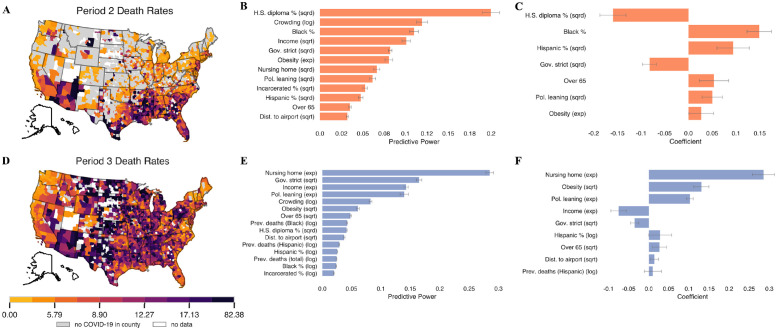
A summary of model results in periods 2 and 3. **(A, D)** distributions of death rates in period 2 and 3, respectively. **(B-C)** feature importance and coefficients for the random forest and LASSO model, respectively, when predicting period 2 death rates. **(E-F)** the same plots for period 3 death rates. Any variables that have a coefficient of zero in the LASSO model are excluded from the plot. Error bars indicate one standard deviation (random forest models) and one standard error (LASSO models). Counties are plotted using the U.S. Census Bureau’s 2019 shapefiles [24].

In our most robust tests, we include a variety of other variables of interest and run multiple machine learning models, the results of which are contained in Fig 5.

In period 1, we focus mainly on virus introduction because the disease was new to the country and virus spread was mainly associated with how early the disease was introduced in a county (S3 Fig). From any of the models presented in S4 Fig, we see that distance to a major airport is one of the strongest predictors of being exposed in period 1. The coefficient on this variable is negative, suggesting that counties farther from a major airport are less likely to be exposed. S4 Fig also indicates that democratic counties are more likely to be exposed to the virus in period 1.

In period 2, we find that counties farther from airports are more likely to be exposed to the virus for the first time (see S5 Fig). Other variables such as nursing home population and income are more important in determining virus introduction in period 2. We find that education and race/ethnicity are the strongest predictors of COVID-19 severity in period 2 (Fig 5B and 5C). We also observe the positive coefficient on political leaning and the strong negative coefficient on governor stringency (Fig 5C), suggesting that Republican counties and counties with less strict mandates fared worse in this period. The magnitude of these coefficients (and predictive power in the random forest model) is less than that of education and percent Black. See S3–S5 Figs for more observations and analysis on virus introduction and severity in periods 1 and 2.

In period 3, we focus mainly on virus spread because the virus was introduced in nearly all counties before October 2020. The top predictors of mortality in all models in period 3 are some combination of nursing home population (consistently the best predictor), income, education, crowding, political leaning, and governor stringency (Fig 5E and 5F). The random forest model more readily picks out mask-wearing (S6 Fig). Political leaning has a positive coefficient in all models, as shown in Fig 5E and 5F, suggesting higher death rates in Republican areas, even when controlling for other potential confounders. Political leaning is also an important predictor, particularly in the random forest model (Fig 5E). S6 Fig shows that preventative behaviors are important, but political leaning has a greater predictive power than either mask-wearing or mobility. When we add these behavioral variables into the model, the predictive power of political leaning and governor stringency decreases in both models. Still, in the random forest model, they remain the second and fourth most predictive explanatory variables, respectively (see S6 Fig). Similarly, in the LASSO model, political leaning still has the second-largest coefficient after adding behavioral variables to the model. This reduction provides evidence that the effect of political differences is partially driven by behaviors.

## Discussion

In this study, we conducted analyses to investigate the relationships between politics, socio-economics, vulnerabilities, behavior, and county-level COVID-19 outcomes. Our goal was to develop an understanding of the relationships between demographic, political, and social vulnerabilities between February 2020 and February 2021, with a focus on understanding the heterogeneous impact of COVID-19 during the winter months, during which over 249,000 of the 462,000 deaths occurred. To accomplish this, we first developed an understanding of the relationship between political leaning and COVID-related deaths across the three periods, motivated splitting the year into three periods using k-means clustering, and conducted a preliminary descriptive bivariate analysis that showed clear trends across political leaning, income, household crowding, and population density when accounting for the stringency of governor mandates. Democratic, high-income, high-crowding, and high-density counties were all associated with lower death rates during the winter months. To rigorously test these trends, we conducted a regularized multiple variable regression analysis using both linear models.

In the first period–February to May 2020–when cluster 1 first peaked, testing was limited and research on how to best mitigate disease transmission was in its early stages. During this time, the pandemic took hold in highly-populated, internationally well-connected urban areas, particularly in the Northeast and the Seattle area. In this period, counties where virus introduction took place earlier had greater disease transmission and higher numbers of deaths.

By the second period, which spans the summer months from June to September 2020, research had firmly established the importance of mask-wearing, social distancing, and other non-pharmaceutical interventions in mitigating COVID-19 transmission. However, the disease spread to new areas in the South of the US and many rural counties experienced their first COVID-19 outbreak.

By period three, which spans the winter months (October 2020–February 2021) and includes the nationwide most intense period of COVID-related deaths (early January), the disease had been intensely circulating in the population for nearly eight months, and there was ample and well-documented evidence on the effectiveness of non-pharmaceutical interventions in reducing disease transmission. Despite the available resources and knowledge gained in periods 1 and 2, Fig 2A shows that the country experienced over half of its deaths in period 3. However, the impacts of the disease were not geographically uniform. In fact, counties in certain areas were able to better prevent deaths than others. Strikingly, we found that the median death rate of counties with strongest Republican political leaning is between 40% and 300% greater than the median death of counties with the strongest Democratic political leaning depending on the stringency of governor interventions (Fig 4). Even after controlling for a diverse array of social vulnerabilities, the importance of political leaning in predicting death rate either doubled or tripled depending on the type of model from period 2 to period 3 (Fig 5E and 5F).

We note several shortcomings still present in our investigation approach. First, while it allows us to separate three distinct periods of the pandemic, this type of analysis is not well-suited to assess the effect of population-level behavioral changes –such as mask-wearing and social distancing– on the trajectory of the COVID-19 pandemic, since these potential changes of trajectory happen in finer temporal scales. In other words, summarizing approximately three months’ worth of mask-wearing or mobility washes out critical week-to-week fluctuations in these variables. However, other studies have overcome this limitation by using different methods to make the connection between behavior, politics, and disease outcomes [2, 4, 27]. Second, we are limited by certain shortcomings in our data sources: sparsity in the mask-wearing data and the potential reflection of COVID-19 related effects in our data: the nursing home data used in this analysis is from 2021 and might intrinsically contain COVID-19 related effects due to the large number of deaths among elderly individuals across the pandemic. Moreover, our income variables are considered constant over time and do not incorporate the shocks in income change caused by the pandemic.

Despite these shortcomings, our findings should raise concern regarding the effects of political leaning and political leadership on the mortality due to COVID-19 and the social and economic spill-overs of the impacts of deaths on the families and communities affected. We require further research to conclusively determine why political leaning and political leadership remain strong predictors of COVID-19 mortality rates, beyond the associations with behavior and preventative factors we included. As of winter 2022, political affiliation remains a strong predictor of vaccine refusal by individuals and state vaccination in relation to who is Governor [28, 29]. It is essential, for reasons of accountability and preventing future preventable deaths, for public health research to document the impact of political viewpoints and agendas on the spread of the pandemic in the US.

## Supporting information

S1 Text Additional data and methodology details. (PDF)

S1 Table Variables and corresponding quantiles for Fig 4. All cutoffs are determined using quantiles such that 20% of counties fall in each group.
(XLSX)

S2 Table The number of counties that have been exposed to the virus in each quantile shown in Fig 4. In period 1, certain groups have low counts (i.e. population density qunatiles for loose and middle governors). By period 3, all groups have at least 45 counties.
(XLSX)

S3 Table A summary of model performance in predicting spread across the three periods. We choose to evaluate model performance on *R*^2^ of residuals as well as the Mean Absolute Error. We also include the Moran’s *I* as a measure of the spatial autocorrelation. High *R*^2^, low MAE, and low Moran’s *I* are desirable, suggesting that the Random Forest consistently performs the best of all three models.
(XLSX)

S1 Fig A summary of variables considered. We include variables that we believe could impact COVID-19 outcomes, as depicted in this conceptual framework. For certain variables (marked with †) such as occupation, we do not have data to directly measure the effects of the variable but include other variables that serve as proxies, such as education and income in the case of occupation. For other variables (marked with **), we have limitations of data and thus we exclude in some parts of the analysis. For example, we only have county-level mask-wearing data in period 3. The domains are derived from the World Health Organization’s framework for action on the social determinants of health [30].
(EPS)

S2 Fig Proportion of counties exposed to COVID-19 and median deaths rate for six population variables broken down by period and the stringency of governor’s interventions. (**A**) In period 1, the majority of counties that are exposed to COVID-19 are Democratic and highly populated. By period 2 and especially period 3, most counties have been exposed to COVID-19. (**B**) Across all six variables, the death rate in period 3 is the highest while the death rate in period 1 is the lowest, suggesting that outbreaks worsened throughout the year. General trends observed in periods 1 and 2 flip in period 3. Certain variables show clear differences between Democratic and Republican governors, such as period 2 political leaning trends.
(EPS)

S3 Fig Coefficients of a LASSO model that attempt to predict spread in period 1. The day a county is seeded emerges as the clear strongest predictor. As a result, we focus mainly on virus introduction in period 1. Any variables with a coefficient of zero are excluded from the figure. Error bars indicate one standard error.
(EPS)

S4 Fig A summary of virus introduction model runs in period 1. **(A)** Variable coefficients after running a logistic regression, excluding both distance to a major airport and crowding. The model accuracy is 0.79, sensitivity is 0.81, and specificity: 0.69. **(B)** Same results including distance to a major airport and crowding. The model accuracy in this case is 0.84, the sensitivity is 0.86, and the specificity is 0.79. The coefficient on political leaning changes from -7.20 to -2.30. Error bars indicate one standard error.
(EPS)

S5 Fig Coefficients of a logistic regression predicts whether a county is first introduced to the virus in period 2. The model accuracy is 0.69, the sensitivity is 0.71, and the specificity is 0.56. As indicated by the positive coefficient on the political leaning variable, more Republican counties are introduced to the virus in period 2. Error bars indicate one standard error.
(EPS)

S6 Fig A summary of our virus severity model runs in period 3, including mask-wearing and mobility (as a proxy for social distancing). **(A-B)** show the result of the random forest and LASSO models, respectively, when we exclude the behavioral variables from the analysis. **(C-D)** shows the same results, this time including behavioral variables. The feature importance for political leaning in the random forest model decreases from 0.18 to 0.13 when adding behavioral variables. The feature importance for governor stringency remains at 0.085 across both model runs. The coefficients of political leaning decreases from 0.36 to 0.35 in the LASSO model, while the coefficient of governor stringency does not change from -0.12. Error bars indicate one standard deviation (random forest models) and one standard error (LASSO models).
(EPS)

S7 Fig A histogram of the number of international flights from the 250 airports covered by the US Department of Transportation. We observe three modes in this histogram and choose to classify large international airports as the airports in the top third of international air traffic.
(EPS)

S8 Fig A summary of the Spearman’s Rank-Order correlation (*r*_*s*_) between variables. Only variables for which there was a correlation with another variable of absolute value greater than 0.5 appear in this figure. **(A)** Correlations when including all counties, as is the case with virus introduction analyses. **(B)** Correlations when including only counties that have been seeded in period 1 or 2, as is the case with period 2 spreading analyses. **(C)** Correlations when excluding only counties that were not seeded at any point in the year, as is the case with period 3 spreading analyses. **(D)** Correlations when including only counties with available period 3 mask usage data.
(EPS)

S9 Fig Elbow plot from performing k-means clustering on county-level time series. We first normalize the each county’s time series of COVID-19 deaths using z-scores. We then cluster the normalized time series using k-means clustering with a Euclidean distance metric. This elbow plot tells us that a logical choice for *k* is anywhere between *k* = 2 and *k* = 5. In this work, we have chosen *k* = 3.
(EPS)

S10 Fig A summary of counties with mask-wearing data. **(A)** shows public mask-wearing rates as percentage of people who self-describe as wearing a mask most or all of the time in public. We do not have mask-wearing data for counties that are colored white. **(B)** A comparison of counties with and without mask usage data. We see that the counties with mask usage data have generally lower death rates. Counties are plotted using the U.S. Census Bureau’s 2019 shapefiles [24].
(EPS)

S11 Fig CAR poisson model non-spatial parameter estimates for periods 2 and 3. (EPS)

S12 Fig CAR poisson model Spatial model component during periods 2 and 3. Counties are plotted using the U.S. Census Bureau’s 2019 shapefiles [24].
(EPS)

S13 Fig Traceplots for non-spatial parameters in CAR poisson model. (EPS)

S14 Fig Convergence diagnostics for all parameters in CAR poisson model. (EPS)

S15 Fig A summary of our virus spread model runs across all periods for the spatial lag model. Hatched bars indicate the indirect effects of a given variable (i.e., if county 1 is near county 2, the performance in county 1 affects county 2 and vice versa). **(A)** shows the coefficients in period 1. **(B)** shows the same same for period 2. **(C)** shows the same for period 3, including counties without mask data. Finally, **(D)** is the results when including only counties with mask data. Variables with a coefficient of zero are not included. Error bars indicate one standard error of the direct coefficients.
(EPS)

S16 Fig Out of sample predictions of death rates for the random forest model for five random train-test splits in each period. **(A)** show the results for period 1 and **(B)** and **(C)** show the results for period 2 and 3, respectively. The random forest models perform well in all three periods, with low mean absolute error, suggesting that the model is not over-fitting.
(EPS)
