# Supplementary material for: The evolving roles of US political partisanship and social vulnerability in the COVID-19 pandemic from February 2020–February 2021
Source: PLOS Glob Public Health. 2022 Dec 5;2(12):e0000557. doi: 10.1371/journal.pgph.0000557 (PMC10021880; doi:10.1371/journal.pgph.0000557)
Supplement: S1 Text — (PDF) [file pgph.0000557.s001.pdf]

# The evolving roles of US political partisanship and social vulnerability in the COVID-19 pandemic from February 2020 - February 2021: Supporting Information

Justin Kaashoek[1,2], Christian Testa[3], Jarvis T. Chen[3], Lucas M. Stolerman[2,4,5], Nancy Krieger[3], William P. Hanage[6], Mauricio Santillana[2,6*]

**1** Harvard College, Cambridge, Massachusetts, United States of America
**2** Machine Intelligence Group for the Betterment of Health and the Environment, Network Science Institute, Northeastern University, Boston, Massachusetts, United States of America
**3** Department of Social and Behavioral Sciences, Harvard T.H. Chan School of Public Health, Boston, Massachusetts, United States of America
**4** Department of Pediatrics, Harvard Medical School, Boston, Massachusetts, United States of America
**5** Department of Mathematics, Oklahoma State University, Stillwater, Oklahoma, United States of America
**6** Department of Epidemiology, Harvard T.H. Chan School of Public Health, Boston, Massachusetts, United States of America

* msantill@g.harvard.edu

## Methods

Here we present a summary of our data sources and methods. We provide details on pre-processing for data from Johns Hopkins' daily death counts, the American Community Survey, the Center for Medicare and Medicaid Services, the VERA Institute, the Center for Disease Control's PLACES dataset, the US Department of Transportation, the Oxford COVID-19 Government Response Tracker, the New York Times, Delphi Epidata, Google, and The COVID Tracking Project. We then provide details on our clustering and modeling methodology.

## Data Sources

The section presents the data sources and caveats for the variables studied in this analysis.

### Response Variable

County-level COVID-19 death is provided by the Center for Systems Science and Engineering at Johns Hopkins University's publicly available data repository [1]. We limit our data to the 50 states and Washington D.C. We also apply a 7-day average to smooth the daily death counts to account for noise in the data.

### American Community Survey

We obtain race/ethnic composition, education, age, income, population density, and household crowding from 2019 5-year estimates of the US Census Bureau American Community Survey (ACS) [2]. We break down race/ethnicity by percent white (not

Hispanic), Black, Asian, Hispanic, and other. Each white non-Hispanic, Black non-Hispanic, Asian non-Hispanic, and Hispanic percentage indicate estimates for the number of individuals who are precisely one race/ethnicity.

We calculate education as the percent of the population with at least a high school diploma. A high "education" value indicates a highly educated county. We also break down age by percent of the population under the age of 19 and the percent of the population over the age of 65. Income represents the median household income of a county, and population density is calculated by dividing a county's population by its available land area, as provided by the Census Bureau. Finally, household crowding is also provided by the ACS and is the estimation of the number of households with more people than rooms.

We note that the values of all variables from the ACS are considered constant across the pandemic and were gathered before COVID-19 hit the US. This means, for example, that our income variable does not account for income shocks that occurred as a result of the pandemic.

### Center for Medicare and Medicaid Services

We obtain nursing home data from the Centers for Medicare & Medicaid Services, which provide data for the average number of nursing home residents per day, organized by provider [3]. We divide this number by a county's population to determine the percentage of the population that is in a nursing home. These data were most recently updated in February 2021. As a result, the data might reflect the impact that COVID-19 has had on the elderly population.

### VERA Institute

We obtain incarceration data from the VERA Institute of Justice that determine jail and prison populations at the county-level [4]. We elect to use only the jailed population to proxy the incarcerated population because prison data was only available in all states in 2014. In contrast, jail data has been available since 2018. Using incarceration data that is more than five years old might not reflect the currently incarcerated population. Delaware, Rhode Island, Vermont, and Connecticut do not have local jails, and therefore, do not participate in the Bureau of Justice Statistics jails data collection [4]. We assume that these states do not have any jailed population.

### PLACES dataset

We obtain obesity data from the CDC's PLACES dataset which is derived from the Behavioral Risk Factor Surveillance Survey [5]. Obesity is calculated as the percentage of the adult (18 years or older) obese population.

### US Department of Transportation

Air Travel data is obtained from the US Department of Transportation and contains the scheduled international passenger traffic every year [6]. We average the provided quarterly numbers from 2019, yielding data on 250 airports. We then classify an airport as a *major international airport* if it falls within the top third of airports with the most international passenger traffic. We choose this threshold because the histogram of international flights from the 250 airports is tri-modal (S7 Fig). We calculate the distance to a major airport as the straight-line distance from the center of a county, as provided by Johns Hopkins, to the closest major airport. If a county contains a major airport, this distance is zero.

## Oxford COVID-19 Government Response Tracker

We determine governor stringency using the Oxford COVID-19 Government Response Tracker (OxCGRT) [7]. OxCGRT tracks all government COVID-19 responses and ranks each regulation on a scale from 0 to 100 based on the stringency of the response, where 100 is the most stringent. We calculate the average stringency for all state-wide responses in each period, excluding the last four weeks of the period to account for the lag between governor interventions and deaths [8].

## Election Results

Political leaning is obtained from The New York Times' 2020 election data. We calculate political leaning as the number of individuals who voted for Joe Biden subtracted from the number of individuals who voted for Donald Trump divided by the total number of voters. This calculation results in a value between -1 and 1, where a value of -1 represents a county where all voters voted for Joe Biden, and a value of 1 represents a county where all voters voted for Donald Trump.

## Delphi Epidata

Public mask usage data is obtained from Facebook's COVID-19 symptom survey and is an estimate of the percentage of people who wore a mask for most or all of the time while in public in the five days before filling out the survey [9]. This survey contains only data beginning on September 8, 2020. Because mask usage is known to be a dynamic variable that has changed over the past year, we choose to include only mask usage data for period 3 [10].

Unfortunately, these data are sparse at the county level compared to our other data sources. S10 Fig suggests that counties with these data might not be representative of all counties. Of the counties without mask data, approximately 2000 are Republican, and about 200 are Democratic. The excluded counties, however, tend to have higher death rates.

Because of the limitation of these data, however, we present results both including and excluding counties with mask usage data (Fig 5 and S6 Fig).

## Google Mobility

We use Google's mobility data as a proxy for social distancing and interactions between individuals who do not live together [11]. Google provides these data as percent changes in mobility from a baseline value. The data is separated into retail and recreation, grocery and pharmacy, parks, transit stations, workplace, residential mobility. We average the percent change in workplace mobility from the baseline in each period for counties with available data. We choose to use workplace mobility because this is the least sparse category that we believe still proxies for interactions between individuals not living together. For a robustness check, we also averaged workplace and retail and recreation mobility if counties had both data points available with similar results. If a county did not have both data points available, we used only workplace mobility.

## The COVID Tracking Project

We use The COVID Tracking Project's Racial Data Tracker to obtain state-level estimates of COVID-related deaths broken down by race and ethnicity. Because there are no reliable county-level datasets on COVID-related deaths by race/ethnicity, we multiply the state level estimates by the proportion of a state's Black, Hispanic, or total

population in a given county (using the Census Bureau's state and county population numbers).

## Plotting

All maps are created using the U.S. Census Bureau's 2019 county shapefiles [12]. S12 Fig plots counties using the urbnmapr package in R [13,14], which uses census shapefiles under the hood. All other maps use Matplotlib to directly plot the county shapefiles. S11, S13, and S14 Fig are constructed with R. All other plots are created with a combination of Matplotlib [15], GeoPandas [16], and seaborn [17].

## Clustering

We first convert smoothed daily death data (using a 7-day smoothing) to $z$-scores (mean subtracted from data, divided by the standard deviation) to allow cross-county comparisons. These z-scores are stored in a matrix $\mathcal{A}$ where each row of the matrix contains the standardized time series of death counts in a particular county, and each column represents one day of data. The matrix $\mathcal{A}$ is $3067 \times 382$ since we have 3067 counties and 382 days of data.

We then perform a $k$-means clustering on our data using Python tslearn's TimeSeriesKMeans package (with euclidean distance metric and 10 random initializations) [18–20]. By clustering the data, we can think of the time series for each county as a point in a 382-dimensional space. The $k$-means clustering algorithm can be described as follows: (1) A total of $k$ counties are randomly chosen as the initial cluster centers. (2) All other counties are assigned to one of the $k$ clusters based on their minimum distance to the cluster means. (3) The new mean is then calculated as the centroid of each k cluster. Steps (2) and (3) are repeated until convergence is reached. We use simple Euclidean distance for this analysis, although we also tried other distance metrics such as dynamic time warping, and we obtained similar results.

We select the number of clusters ($k$) using an elbow plot. An elbow plot for $k$-means clustering shows the total distance from each county to its assigned cluster center. Because increasing the number of clusters decreases the total distance, a "kink" in the elbow plot represents a new cluster that does not significantly reduce the total distance. Then, a kink in the elbow plot does not significantly reduce the total distance, thus yielding a reasonable choice for $k$. S9 Fig shows the elbow plot that results from performing $k$-means clustering on $A$. We observe a kink in the plot around $k = 3$ and therefore choose to perform 3-means clustering. To select the optimal clusters, we run 3-means clustering with ten different combinations of counties as the initial three clusters and select the configuration with the smallest total distance from each county to the cluster center.

## Model Details

In this section, we present the model details for our virus introduction and virus spread models.

### Virus Introduction Models

To predict virus introduction, we run a logistic regression without any regularization. We consider the virus to be introduced to a county in a given period if at least five deaths occurred in the county throughout the period. To account for spatial autocorrelation, we include latitude and longitude on all models. We choose to exclude latitude and longitude in our visualization of feature importance and model coefficients

because latitude and longitude are not inherent measures of risk or disease spread. We additionally run a logistic regression with L1 regularization to predict virus introduction as a robustness check.

## Virus Spread Models

**Regressions.** To predict virus spread, we first run two different regressions: a LASSO regression and a random forest regression. The LASSO regression can help select only the most important variables, and the random forest regression captures nonlinearities in the data. To calculate feature importance in the random forest regression, we calculate permuted feature importance. This metric perform a random permutation on a given explanatory variable and calculates the resulting loss in accuracy of the model. To address spatial autocorrelation in these regressions, we include latitude and longitude as predictors in all models.

We run the LASSO regression using Python sklearn [21]. This regression seeks to minimize the following objective function:

$$\frac{1}{2 \cdot N} \cdot ||y - Xw||_2^2 + \alpha \cdot ||w||_1 \qquad (1)$$

where $N$ is the number of data points, $y$ is the observed death rate, $X$ is a matrix of the independent variables, $w$ is a vector of coefficients, and $\alpha$ determines how much to penalize coefficients. A large $\alpha$ will force more coefficients towards zero. We run the LassoCV model with values of $\alpha$ ranging from 0 to 1 with a step size of 0.001 and 5-fold cross-validation. We then weight the regression by the log of a county's population.

The second regression we consider is a random forest regression. To ensure that we find the optimal model that does not over-fit when trained on all of our data, we first fit the model using all variables but 70% of counties as training data. We perform a grid search using 5-fold cross-validation on our training data and select the hyper-parameters that yield the lowest Mean Absolute Error (MAE). The hyper-parameters on which we perform the grid search are the following: (i) number of estimators (either 50, 70, or 90), (ii) maximum number of features (either the number of features or the square root of the number of features), (iii) maximum tree depth (between 5 and 100), (iv) the minimum number of samples required to split a node (2, 5 or 10), (v) and the minimum number of samples required for a leaf node (1, 2 or 4). We toggle bootstrapping on and off as well. We repeat this process three times, which yields an optimal random forest configuration for each period (see S16 Fig for out-of-sample predictions). Once we have these optimal configurations, we fit the models on all counties and predict purely in-sample.

We test four monotonic transformations of our input variables to account for nonlinear relationships between explanatory and response variables: squared, square root, log, and exponential. If any of these transformations yields a higher Pearson's correlation with either death rate or log-transformed death rate than the correlation between the untransformed variable and death rate, we apply the transformation to the given variable. We find the optimal transformation for each variable in each period. In all three time periods, most explanatory variables yield higher correlations to the log of our response variables, so we take the logarithm of our response variable in the remainder of our analysis.

Finally, it is important to distinguish between counties that are included in virus introduction vs. virus severity. In our virus spread models, we consider a county that was either introduced to COVID-19 in the period of interest or a previous period. In our virus introduction models, however, we consider all counties and aim to predict counties first exposed to the virus in the period of interest. For example, for virus spread models in period 2, we only include counties that were introduced to the virus in

period 1 or 2. In running period 2 virus introduction models, we only attempt to predict counties first exposed to the virus in period 2 out of all counties.

**Excluding Variables.** Throughout our regression models, we choose to exclude variables that are measures of a similar idea. Excluding these variables improves the condition number of the matrix of explanatory variables, making our results more trustworthy. The condition number is a measure of how much output can change given a small perturbation in the input [22]. We remove density (as crowding serves as a proxy), percent under 19 (as percent over 65 serves as a proxy), and certain race/ethnicity variables (percent white not Hispanic, Asian, and other, as percent Black and Hispanic serve as a proxy). We keep all obesity, education, and income despite their correlations because these variables address different concepts and vulnerabilities. Removing these variables reduces the condition number of the matrix of explanatory variables from 133.99 to 107.41 for our virus introduction analyses. In period 2 spread analysis, which only includes counties exposed in period 1 or 2, removing these variables reduces the condition number from 150.98 to 119.35. Finally, in our period 3 spread analysis, removing these variables reduces the condition number from 93.61 to 58.20

**CAR Poisson Model.** We also implemented a conditional autoregressive (CAR) Poisson model of COVID-19 county death rates in the United States. The inclusion of a conditional autoregressive spatial component in this model is intended to address how robust the relationships are between observed covariates and COVID-19 death rates, given the expected correlations in rates between counties which neighbor one another.

The implementation of our model is based on the Stan Case Study, *Exact Sparse CAR Models in Stan* [23]. Spatial models, including CAR models and improvements on the Besag-York-Mollié model, have often been used in current epidemiology and disease risk mapping applications to distinguish spatially structured effects from the effects of observed covariates [24,25].

To describe our model, we write the deaths observed as $y_1, y_2, ..., y_{2683}$ for each of the 2,683 counties which have neighboring counties and for which all covariates were available and hence were considered in our main manuscript. Letting $X_i$ for $i$ in $1 \ldots 2{,}683$ represent the vector of observed covariates for the $i$th county and similarly $P_i$ represent the population of the $i$th county, we write that

$$y_i \sim \text{Poisson}(\exp(X_i\beta + \phi_i + \log(P_i))),$$

where $\beta$ is a vector of the estimated coefficients for the covariates and $\phi_i$ is the spatial component of the model. See [23] for the details of the prior distributions on $\beta$ and $\phi$. This model is fit twice with data from periods 2 and 3 separately.

Given the computational complexity in fitting these models with over 2600 county observations and a large number of covariates, we opted only to select the variables that had the highest features importance measures in the LASSO and spatial linear models from our main manuscript. To include parameters parsimoniously, we chose to include parameters that appeared in the top three most important features from the LASSO and spatial linear models for periods 2 and 3. Since we only modeled the probability of counties being seeded during period 1 and did not model death rates, we have only calibrated the spatial model presented here to the deaths data from periods 2 and 3.

All analyses for the CAR Poisson models were conducted using R version 4.0.2 [14] and the model analyses were conducted in the Bayesian statistical computing and modeling framework Stan using the No-U-Turns Hamiltonian Monte Carlo Sampler [26,27].

**Spatial Lag Model.** We also predict virus spread with a spatial lag model that better accounts for spatial auto correlations. We run the spatial lag regression using pysal's ml_lag model [28]. This model attempts to find the maximum likelihood estimate of the following:

$$y = \rho W y + \beta X + \epsilon \quad (2)$$

where $y$ is the median death rate, $X$ is a matrix of independent variables, $\rho$ and $\beta$ are the spatial autoregressive coefficient and independent variable coefficients, respectively, and $Wy$ represent the spatial lag. $W$ is a weight matrix that determines how heavily nearby counties impact a given county. For our analysis, we construct $W$ using $n$ nearest-neighbors, where all $n$ nearest-neighbors are weighted equally. We first run the spatial lag model using all variables with values of $n$ ranging from 1 to 20 and find that $n = 5$ yielded the lowest mean absolute error (MAE).

For all of the models described, we also vary the cutoff at which we consider the virus introduced in a county. We ran the same set of analyses using 1 and 3 as the death cutoff (instead of 5). Finally, we remove the weights on the LASSO regression. All of these robustness checks yield a similar narrative as the one described in our Results.

## Model Results for Periods 1 and 2

In this section, we present the results for virus introduction and virus severity for periods 1 and 2.

### Period 1 (January 26, 2020 - June 2, 2020): Democratic Counties are First Exposed to COVID-19

In period 1, we focus on virus introduction since the disease spread is almost entirely driven by how early a county was exposed to the virus. The coefficients of a LASSO regression model support this and can be seen in S3 Fig.

From any of the models presented in S4 Fig, we see that distance to a major airport is one of the strongest predictors of being exposed in period 1. The coefficient on this variable is negative, suggesting that counties farther from a major airport are less likely to be exposed. We also note that Democratic counties are more likely to be exposed to the virus in this period. S4 Fig shows the tests we run to address whether we observe more virus introduction in Democratic counties due to proximity to international airports. We begin by removing distance to an airport and crowding from the analysis (S4(A) Fig; accuracy: 0.79; sensitivity: 0.81; specificity: 0.69). This results in a coefficient on political leaning of -7.20. Adding distance to an airport back into the model (accuracy: 0.80; sensitivity: 0.82; specificity: 0.73), the coefficient on political leaning reduces in magnitude to -6.26, a 13% change. Finally, we add crowding back into the model (S4(B) Fig; accuracy: 0.84; sensitivity: 0.86; specificity: 0.79), further reducing the coefficient on political leaning to -2.30, an additional 63% decrease. Between distance to an airport and crowding, the coefficient on political leaning has reduced by 68%.

### Period 2 (June 3, 2020 - October 7, 2020): COVID-19 Spreads to Republican Counties

County-level mask-wearing data were not available for periods 1 or 2, and thus we cannot conduct any tests with behavioral variables. As a result, we focus on vulnerabilities, demographics, and political variables.

Counties far from airports are more likely to be exposed to the virus for the first time as the disease spreads to more rural, Republican areas, as shown by S5 Fig (accuracy: 0.69; sensitivity: 0.71; specificity: 0.56). This Fig hows the results of running the same logistic regression as S4(B) Fig, just using virus introduction for period 2 as the response variable. Both the coefficients on political leaning and airport are positive;

however, other variables such as nursing home population and income are more important in determining virus introduction in period 2.

Based on the feature importance and coefficients obtained from the regression analyses, we conclude that among counties that have been exposed to the virus, more severe COVID-19 spread is correlated with indicators of higher social vulnerability (as indicated in the socio-economics and vulnerabilities boxes of S1 Fig). Fig 5A shows a distribution of the death dates across all US counties in period 2 and Fig 5B and Fig 5C indicates which variables have the most predictive power. The top variables in both models are vulnerability-related: education and race/ethnicity.

We also conclude that Republican areas have higher death rates. There may be a significant impact of behavioral variables such as mask usage and social distancing, but we are unable to test these impacts in period 2. Instead, we see that the importance and coefficient on political leaning are small relative to the coefficients on vulnerability-related variables. Therefore, vulnerable populations were significantly impacted by COVID-19, and Republican counties and counties with less strict regulations were hit hardest.

## Results for CAR Poisson Model

We found that the results of fitting a spatial sparse CAR Poisson model were consistent with our findings from the main analyses for periods 2 and 3 (S11 Fig).

These model estimates reflect the associations between county-level covariates and COVID-19 death rates after accounting for the estimated spatial correlation structure included in the model.

We found that higher percentages of residents in nursing homes, Black non-Hispanic population percentages, and Hispanic population percentages at the county level were associated with higher COVID-19 death rates during period 2. Counties with higher high school graduation rates were associated with having lower COVID-19 death rates in period 2 after accounting for spatial correlations. During period 3, counties where the population voted more Republican (positive political lean), and counties with higher percentages of the population living in nursing homes were associated with higher COVID-19 death rates. Counties with higher median income, greater high school graduation rates, and policy strictness during period 3 were found to have lower COVID-19 death rates.

We present the spatial model component visualized as $\exp(\phi_i + \log(P_i))$, the expected COVID-19 deaths per capita in each period conditioning out the effects from covariates, in S12 Fig.

S12 Fig allows us to visualize the estimated spatial correlation structure and how neighboring counties tend to be correlated with one another. In essence, we expect to see that counties with high rates are surrounded by counties with high rates and vice-versa for low rate counties.

Contemporary advice recommends that Bayesian models should be considered to have converged only if the Markov chains have convergence diagnostics of $\hat{R} < 1.05$ [26,29]. In S14 Fig, we present the $\hat{R}$ convergence diagnostics for our non-spatial and spatial effects for both periods 2 and 3, which are all below 1.05.

## Results for Spatial Lag Model

We found that the results of fitting a spatial lag model were mostly consistent with our findings from the main analyses for all periods (S15 Fig). In period 1, the day county is seeded is the strongest predictor of the number of deaths in a county. In period 2, the nursing home population, education, and race/ethnicity are the top three predictors. In period 3, the spatial lag model less readily picks out political leaning, although the

coefficient is still clearly positive. When including mask-wearing data, political leaning is the 6th most predictive variable. In both cases, the nursing home population in a county is the best predictor of the number of deaths the county experiences.

## References


1. Dong E, Du H, Gardner L. An Interactive Web-Based Dashboard to Track COVID-19 in Real Time. Lancet Infect Dis. 2020;20(5):533–534.

2. U S Census Bureau. American Community Survey 5-year Estimates; 2015-2019. Available from: https://www.census.gov/programs-surveys/acs.

3. The Centers for Medicare & Medicaid Services. Provider Information; 2021. Available from: https://data.cms.gov/provider-data/dataset/4pq5-n9py.

4. Vera Institute of Justice. Incarceration Trends Dataset; 2020. Available from: https://github.com/vera-institute/incarceration-trends.

5. Centers for Disease Control and Prevention. Behavioral Risk Factor Surveillance System: prevalence and trends data: overweight and obesity (BMI); 2020. Available from: https://chronicdata.cdc.gov/500-Cities-Places/PLACES-Local-Data-for-Better-Health-Place-Data-202/q8xq-ygsk.

6. Department of Transportation Office of the Assistant Secretary for Aviation and International Affairs. International Report Passengers; 2021. Available from: https://data.transportation.gov/Aviation/International_Report_Passengers/xgub-n9bw.

7. Hale T, Angrist N, Goldszmidt R, Kira B, Petherick A, Phillips T, et al. A global panel database of pandemic policies (Oxford Covid-19 Government response tracker). Nat Hum Behav. 2021;5(4):529–538. doi:10.1038/s41562-021-01079-8.

8. Ferguson N, Laydon D, Nedjati Gilani G, Imai N, Ainslie K, Baguelin M, et al. Report 9: Impact of non-pharmaceutical interventions (NPIs) to reduce COVID19 mortality and healthcare demand. Imperial College London. 2020;doi:https://doi.org/10.25561/77482.

9. Farrow DC, Brooks LC, Rumack A, Tibshirani RJ, Rosenfeld R. Delphi Epidata API; 2015. Available from: https://github.com/cmu-delphi/delphi-epidata.

10. Lazer D, Santillana M, Perlis RH, Quintana A, Ognyanova K, Green J, et al.. The COVID States Project #26: Trajectory of COVID-19-related behaviors; 2021. Available from: https://osf.io/37jbe/.

11. Google. Google Mobility Reports; 2021. Available from: https://www.google.com/covid19/mobility/.

12. U S Census Bureau. 2019 TIGER/Line Shapefiles; 2019. Available from: https://www.census.gov/cgi-bin/geo/shapefiles/index.php.

13. Strochak S, Williams AR, Ueyama K. urbnmapr; 2019. Available from: https://github.com/UrbanInstitute/urbnmapr.

14. R Core Team. R: A Language and Environment for Statistical Computing; 2021. Available from: https://www.R-project.org/.

15. Hunter JD. Matplotlib: A 2D graphics environment. Comput Sci Eng. 2007;9(3):90–95. doi:10.1109/MCSE.2007.55.

16. Jordahl K, den Bossche JV, Fleischmann M, Wasserman J, McBride J, Gerard J, et al.. geopandas/geopandas: v0.8.1; 2020. Available from: https://doi.org/10.5281/zenodo.3946761.

17. Waskom ML. seaborn: statistical data visualization. J Open Source Softw. 2021;6(60):3021. doi:10.21105/joss.03021.

18. Lloyd S. Least squares quantization in PCM. IEEE Trans Inf Theory. 1982;28(2):129–137. doi:10.1109/TIT.1982.1056489.

19. MacQueen J. Some methods for classification and analysis of multivariate observations. Proc of the Fifth Berkeley Symposium on Math Stat and Prob. 1967;1:281–296.

20. Tavenard R, Faouzi J, Vandewiele G, Divo F, Androz G, Holtz C, et al. Tslearn, A Machine Learning Toolkit for Time Series Data. J Mach Learn Res. 2020;21(118):1–6.

21. Pedregosa F, Varoquaux G, Gramfort A, Michel V, Thirion B, Grisel O, et al. Scikit-learn: Machine learning in Python. J Mach Learn Res. 2011;12:2825–2830.

22. Rice JR. A Theory of Condition. SIAM J Numer Anal. 1966;3(2):287–310.

23. Joseph M. Exact Sparse CAR Models in Stan; 2016. Available from: `https://mc-stan.org/users/documentation/case-studies/mbjoseph-CARStan.html`.

24. Lee D, Rushworth A, Sahu SK. A Bayesian localized conditional autoregressive model for estimating the health effects of air pollution: A Bayesian localized conditional autoregressive model. Biometrics. 2014;70(2):419–429. doi:10.1111/biom.12156.

25. Wakefield J. Disease mapping and spatial regression with count data. Biostatistics. 2007;8(2):158–183. doi:10.1093/biostatistics/kxl008.

26. Stan Development Team. Stan Modeling Language Users Guide and Reference Manual; 2021. Available from: `https://mc-stan.org/`.

27. Homan MD, Gelman A. The No-U-Turn Sampler: Adaptively Setting Path Lengths in Hamiltonian Monte Carlo. J Mach Learn Res. 2014;.

28. Rey SJ, Anselin L. PySAL: A Python Library of Spatial Analytical Methods. Reg Stud. 2007;37(1):5–27.

29. Vehtari A, Gelman A, Simpson D, Carpenter B, Bürkner PC. Rank-Normalization, Folding, and Localization: An Improved Rˆ for Assessing Convergence of MCMC (with Discussion). Bayesian Anal. 2021;16(2). doi:10.1214/20-BA1221.
